# Inpatient forensic psychiatric care: Legal provision in European countries

**DOI:** 10.1192/j.eurpsy.2021.205

**Published:** 2021-08-13

**Authors:** B. Völlm

**Affiliations:** 1 Forensic Psychiatry, University of Rostock, Rostock, Germany; 2 Klinik Für Forensische Psychiatrie, Universitätsmedizin Rostock, Rostock, Germany

## Abstract

**Abstract Body:**

Forensic psychiatry is a specialty of psychiatry primarily concerned with individuals who have either offended or present a risk of doing so, and who also suffer from a psychiatric condition. These mentally disordered offenders(MDOs) are often cared for in secure psychiatric environments or prisons. However, the organisation of these services differs greatly between countries due to different traditions and legal frameworks. Some countries, e. g., require absent or reduced criminal responsibility (at the time of the index offence) in order to enter forensic services while others determine access on the basis of current need for treatment. Numbers detained in forensic services also vary significantly as does length of stay, raising significant economic and ethical challenges. This talk will present different legal concepts determining admission to forensic-psychiatric services, data on length of stay as well as approaches to risk assessment and treatment in Europe.

**Disclosure:**

No significant relationships.

